# A longitudinal investigation of childhood communication ability and adolescent psychotic experiences in a community sample

**DOI:** 10.1016/j.schres.2016.03.005

**Published:** 2016-05

**Authors:** Sarah A. Sullivan, Linda Hollen, Yvonne Wren, Andrew D. Thompson, Glyn Lewis, Stan Zammit

**Affiliations:** a Centre for Academic Mental Health, University of Bristol, Oakfield House, Oakfield Grove, Bristol, UK; b CLAHRC West, Whitefriars, Lewins Mead, Bristol, UK; c Centre for Child and Adolescent Health, University of Bristol, Oakfield House, Oakfield Grove, Bristol, UK; d Bristol Speech and Language Therapy Research Unit, North Bristol NHS Trust, Frenchay Hospital, Bristol, UK; e Division of Mental Health and Wellbeing, University of Warwick, Coventry, UK; f Division of Psychiatry, University College London Charles Bell House, Gower St, London, UK; g Institute of Psychological Medicine and Clinical Neurosciences, University of Cardiff, Haydn Ellis Building, Maindy Road, Cardiff, UK

**Keywords:** Pragmatic language, Expressive speech and language, Adolescent psychotic experiences, Adolescent depression, ALSPAC

## Abstract

**Background:**

Some childhood speech and language impairments precede psychosis but it is not clear whether they also precede adolescent psychotic experiences and whether this association is specific to psychotic experiences.

**Methods:**

Pragmatic language and expressive speech and language (parent-assessed using the Children's Communication Checklist) at age 9 and psychotic experiences and depression at ages 12 and 18 were investigated in 7659 participants from the Avon Longitudinal Study of Parents and Children. Associations were investigated using multivariate modelling.

**Results:**

Poorer pragmatic language at 9 years was associated with psychotic experiences at both ages (12 years OR 1.22, 95% CI 1.11, 1.34; 18 years OR 1.25, 95% CI 1.10, 1.41) but only with depression at 18 years (OR 1.10, 95% CI 1.00, 1.22). Poorer expressive speech and language ability was not associated with psychotic experiences or depression at either age. There was evidence that pragmatic language was specifically associated with psychotic experiences at age 12 but no evidence that the strength of any of the associations changed over time.

**Conclusions:**

Deficits in pragmatic language precede early and late adolescent psychotic experiences and early adolescent depression. Interventions aimed at helping children improve pragmatic language skills may reduce the incidence of adolescent psychopathology and associated psychological disorder and dysfunction later in life.

## 1 Introduction

Psychotic experiences (PEs) are attenuated psychotic phenomena, similar to those experienced by those with a clinical disorder, and occurring in healthy populations. They have a prevalence of between approximately 3 and 30% in community samples (van Os et al., 2009) and are part of the continuum of psychosis (Linscott and van Os, 2010, van Os et al., 2009). Although these experiences are transient and harmless for many, they are also important because they are persistent for some, predictive of later psychotic disorder and are associated with dysfunction (Zammit et al., 2013) and disorders such as depression (Dhossche et al., 2002, Kounali et al., 2014, Scott et al., 2009a, Sullivan et al., 2014, van Rossum et al., 2011).

There is a rich literature on the association between schizophrenia and language (Crow, 2000). Childhood speech and language difficulties e.g. with using language in a social context and speaking in a fluent and intelligible way are associated with adult psychosis (Bearden et al., 2000, Green, 1996, Jones et al., 1994, Welham et al., 2009). However, it is unclear from the current evidence whether antecedent speech and language problems are in expressive speech and language (i.e. the use of sounds, words and sentences at an age appropriate level) or pragmatic language (i.e. the use of language in socially appropriate ways).

Poor pragmatic language (PL) has been defined as “verbosity, excessive topic switching, tendency to dominate verbal interactions, poor adjustment to listener's prior knowledge and limited application of inference” (Adams et al., 2012). A child with poor pragmatic language ability would find it difficult to use language that is appropriate to the social context. Social cognitive ability is required for effective pragmatic language use since, in order to understand the social context of interactions, an understanding of the mental state of the conversational partner is required (Mazza et al., 2008). There is convincing evidence of associations between social cognitive deficits or bias and both psychosis (Clemmensen et al., 2014, Sprong et al., 2007) and PEs (Polanczyk et al., 2010) and, to a lesser extent, depression (Weightman et al., 2014), although the associations with depression are weaker. Pragmatic language problems have been described as a type of social communication disorder and the terms are often used interchangeably. Pragmatic language problems are also a feature of high-functioning autism, however difficulties with pragmatic language alone is not sufficient for a diagnosis of autism since there are other symptoms which are also characteristic of this condition, such as repetitive and stereotyped behaviour (Adams et al., 2012, Law et al., 2015).

Expressive speech and language (ESL) problems, e.g. speech sound substitutions, low intelligibility, reduced vocabulary, difficulty with producing complex sentences and using inappropriate tenses, may be due to verbal memory deficits which are common in psychosis (Keefe et al., 2006). Prospective evidence for the association between antecedent ESL problems and schizophrenia is inconsistent. One study (Bearden et al., 2000) of speech-pathologist assessed ESL at age 7 found strong evidence of an association with schizophrenia in adulthood. However, another study (Cannon et al., 2002) which assessed ESL and receptive language, using a trained psychometrist and a standardised protocol, reported that ESL problems at 3 and 5 years did not predict schizophreniform disorder in adulthood in a cohort of 976 participants. A further study (Reichenberg et al., 2002) of 16 and 17 year old Israelis eligible for military service (n = 635) collected assessments of fluency and speech quality rated by a trained assessor, also found that impaired ESL did not predict a diagnosis of schizophrenia.

There has been only one prospective study of antecedent language problems and PEs in community samples (Cannon et al., 2002) which did not find an association between ESL problems at 3 and 5 years and PEs at 11 years. The authors are not aware of any study to investigate antecedent PL problems and later PEs.

The strong overlap between PEs and depression (Dhossche et al., 2002, Kelleher et al., 2012) means that any association between poor childhood speech and language and adolescent PEs may not be specific but a result of co-morbidity with depression.

The majority of evidence to date suggests that poor childhood ESL is not associated with later schizophrenia. It is possible however that ESL problems are more likely to be associated with depression since there is cross-sectional evidence (Conti-Ramsden and Botting, 2008, Irwin et al., 2002, Maggio et al., 2014) and one prospective study (Bornstein et al., 2013) reporting associations, although others have not found an association (Cannon et al., 2002). There is convincing evidence (Drury et al., 1998, Green and Leitman, 2008) of poor social cognitive ability in schizophrenia suggesting that pragmatic language deficits which reflect difficulties in social cognitive ability may also precede PEs. The evidence of an association between social cognition and depression is much weaker and mostly for clinical depression rather than for sub-clinical depressive symptoms recorded in the general population cohort used for this study. Our primary hypothesis is that PL problems in childhood will be associated with PEs but not depression at 12 and 18 years and that ESL problems will be associated with depression but not PEs at 12 and 18 years. As a secondary analysis, within psychopathologies, we will investigate whether the associations are stronger at 18 years when compared with 12 years.

Prospective birth cohort data on ESL and PL and repeated measures of PEs and depression in adolescence provided the opportunity to investigate these associations. Unfortunately there were no data available on receptive language. Multivariate probit modelling is a technique that allows for simultaneous but separate modelling of more than one outcome in order to directly compare the strength of associations whilst allowing for covariance between outcomes. This method will allow examination of whether communication ability, as assessed by ESL and PL are specific risk factors or whether they infer added risk of both PEs and depressive symptoms.

The authors are not aware of any previous studies using this method to investigate the prospective association between speech and language ability and PEs.

## 2 Methods

### 2.1 Avon Longitudinal Study of Parents and Children (ALSPAC)

The study sample consists of ALSPAC (http://www.bristol.ac.uk/alspac/) participants (Boyd et al., 2012, Fraser et al., 2013). The study website contains details of all the data that is available through a fully searchable data dictionary http://www.bris.ac.uk/alspac/researchers/data-access/data-dictionary//.

The sample is representative of those born at that time in the former county of Avon during this period (Golding et al., 2001).

### 2.2 Ethics

Ethical approval for the study was obtained from the ALSPAC Ethics and Law Committee and the Local Research Ethics Committees.

### 2.3 Dataset

We used a subsample of (n = 7659) the ALSPAC cohort who had provided data on *either* PEs *and*/*or* depression at 12 or 18 years *and*/*or* childhood speech and language ability at age 9 *and* data on at least one of the confounding variables. Missing outcome and risk factor data was imputed (see Section 2.7).

### 2.4 Outcome measures

#### 2.4.1 PEs at 12 and 18 years

The Psychosis-Like Symptom interview (PLIKSi) (Horwood et al., 2008a) is a semi-structured instrument that uses the principles of standardised clinical examination developed for the Schedule for Clinical Assessment in Psychiatry (SCAN) (Wing et al., 1990). It includes 12 ‘core’ questions eliciting key PEs. At 18 years participants were asked about their experiences since their 12th birthday, whereas at 12 years participants were asked about experiences since their last birthday. If an experience was rated as suspected or definite the participant was asked whether the experiences reported were always attributable to the effects of sleep (hypnopompic or hypnogogic experiences), fever, or substance use (occurring only within 2 h of intoxication with drugs or alcohol). If this was the case the experience was rated as not present. For the purposes of this analysis a binary variable was used (no, versus suspected or definitely present). This was necessary due to the distribution of this variable and the fact that the majority of respondents did not report any experiences.

At the 18 year assessments the average kappa value for inter-rater reliability across the time-period of data collection was 0.83 which is considered high using standard benchmarks (Zammit et al., 2013). At the 12 year assessments the inter-rater reliability across all interviewers was ‘very good’ (Kappa = 0.72) according to standard benchmarks (Horwood et al., 2008a).

#### 2.4.2 Depression at 12 and 18 years

Depression was measured using the Moods and Feelings Questionnaire (MFQ) (Angold et al., 1995a) at both ages. At 12 years the questionnaire was parent-rated and at 18 years it was self-rated. Since depression can be considered a continuum the depressive symptom scores were dichotomised to indicate the presence of depression (Kounali et al., 2014). A cut off at a score of 11 or above has been previously used (Joinson et al., 2013). Dichotomisation also allowed the joint modelling with PEs as a binary variable.

### 2.5 Childhood pragmatic language and expressive speech and language

#### 2.5.1 Children's Communication Checklist (CCC) at 9 years (Bishop, 1998)

The CCC was parent-rated on behalf of the child and consists of 53 statements grouped into 7 domains; intelligibility and fluency; syntax; inappropriate initiation; coherence; stereotyped conversation; use of conversational context; conversational rapport. For the purposes of this analysis the *intelligibility and fluency* and *syntax* domains were summed to create a total ESL score.

The *inappropriate initiation*, *coherence*, *stereotyped conversation*, *use of conversational context* and *conversational rapport* domains were summed to create a PL score.

The scores on the speech and language variables were reversed so that higher scores represented worse ability.

### 2.6 Potential confounders

We selected potential confounders from knowledge of PEs, childhood communication disorder and confounders used in similar research. Both gender and socio-economic status are associated with prevalence of PEs (Horwood et al., 2008b), therefore two proxy measures of socio-economic status (maternal education level and marital status at the child's birth) were selected. Additionally, it was likely that concurrent mood would impact on language ability, therefore parent-reported concurrent depression at age 9, collected using a postal questionnaire (Moods and Feelings Questionnaire-Shortened) (Angold et al., 1995b), was included. IQ is associated with both PEs (Horwood et al., 2008a) and language ability. For this analysis only non-verbal IQ was included since there was likely to be co-linearity between verbal IQ and language ability. IQ was assessed at age 8 with alternate items from the Wechsler Intelligence Scale for Children (Wechsler et al., 1992). Those with a non-verbal IQ score of ≤ 70 (n = 53) were excluded due to the difficulty of assessing speech and language ability in this group. Language ability is likely to be affected by autistic traits and this research group has already identified an association between PEs and autistic traits in this cohort (Sullivan et al., 2013). Total autistic factor score across seven autistic traits was used for this analysis (Steer et al., 2010).

The same set of confounders was used in each probit model.

### 2.7 Missing data

In common with similar cohorts there were large amounts of missing data at follow-up. Previous ALSPAC studies (Sullivan et al., 2014) have found that participants lost to follow-up were more likely to have mental health problems and come from lower socio-economic groups. Therefore an analysis which included only those who had remained in the cohort is likely to be biased. We therefore imputed missing data including a broad range of variables associated both with missingness and with missing risk factors or outcomes.

Missing data patterns were examined. Multiple imputation in the Stata statistical package (StataCorp, 2009) was used to impute data using multiple imputation by fully conditional specification using chained equations (Little and Rubin, 2002, van Buuren et al., 1999). One hundred datasets were imputed using auxiliary variables to make the basic assumption (data missing at random) underlying multiple imputation more realistic (further information available on request).

### 2.8 Statistical analysis

Four dimensional (i.e. PEs at 12 and 18 and depression at 12 and 18) probit models (Ntzoufras et al., 2003) were constructed to investigate associations between PL, ESL and each outcome. Due to the non-normal distributions of PL and ESL the reversed raw scores were transformed to z scores using the inverse normal function, before being entered into the probit models.

For our primary analysis we investigated differences between the strength of associations between psychopathologies using likelihood ratio tests which compared a model which allowed estimates between each risk factor (PL and ESL) and each outcome (PEs and depression at 12 and 18 years) to differ compared to a model where the time-points were constrained to be equal. Wald tests were used to test the null hypothesis that the unconstrained and constrained models were equal.

For our secondary analysis we investigated the strength of the association differences by comparing the unconstrained model with one which constrained each psychopathology to be equal within time-points. Again Wald tests were used to test the null hypothesis that the unconstrained and constrained models were equal.

Confounders were added to each model.

Estimates of association in probits were converted into odds ratios to improve interpretability by exponentiating the estimate after multiplying it by 1.6 (Amemiya, 1981, Ntzoufras et al., 2003).

## 3 Results

### 3.1 Descriptive data for participants

Table 1 describes the proportion of missing data for each variable and compares imputed to observed data. The differences between the observed and imputed data were small.

Fourteen percent of participants had suspected or definite PEs and 5.3% were depressed at 12 years. At 18 years 9.4% had suspected or definite psychotic experience and 18.8% were depressed. At 12 years 31.5% of those with depression also had PEs and at 18 years 18.9% of those with depression also had PEs.

### 3.2 Primary analysis

#### 3.2.1 Association between speech and language ability at age 9 and PEs and depression at ages 12 and 18

Table 2 shows the unadjusted association between PL and ESL at age 9 and potential confounding variables and PEs and depression at 12 and 18 years. The likelihood ratio test for common effects across psychopathology is shown. Table 3 shows the adjusted associations.

##### 3.2.1.1 PL

**Fig. 1:**
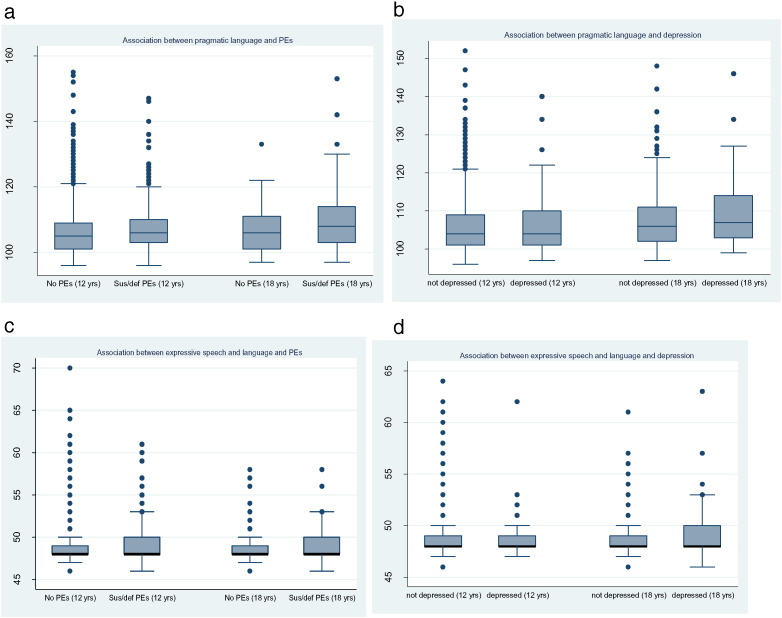
a and b: Association between pragmatic language scores and adolescent psychopathology. c and d: Association between expressive speech and language scores and adolescent psychopathology.

Fig. 1a and b shows a box plot of PL scores against PEs and depression at both ages. Fig. 1a shows that those with PEs at ages 12 and 18 had a higher (poorer ability) median PL score, although this is more marked at 18 years. Fig. 1b shows little difference in PL scores at 12 years between those who were and were not depressed and a small increase in PL scores at 18 years in those who were depressed compared to those who were not.

**Fig. 2:**
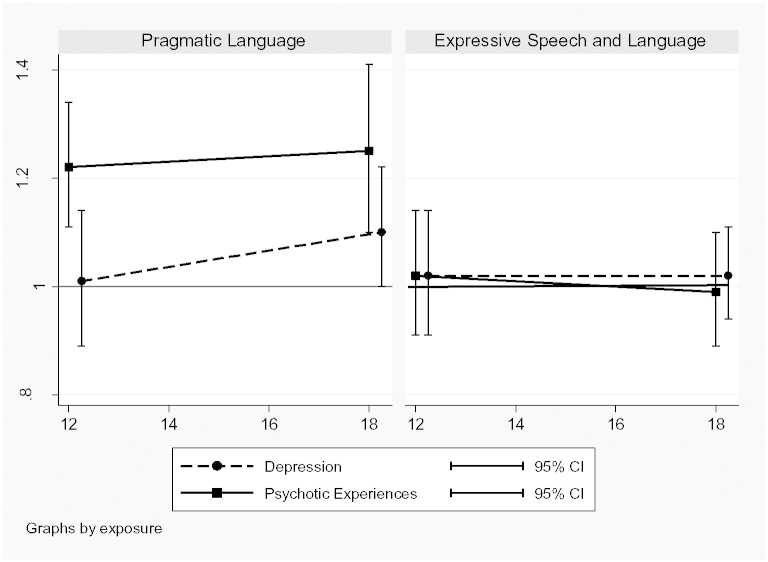
Association (ORs and 95% CI) between speech and language skills at age 9 and psychopathology at ages 12 and 18 years.

There was no evidence of an association between PL problems and depression at age 12 and marginal evidence of an association at age 18, when a 1 SD increase in PL score was associated with a 10% increase in the odds of depression. However, there was stronger evidence of an association between PL problems and psychotic experiences with a 1 SD increase in PL score being associated with a 22% increase in the odds of PEs at 12 years and a 25% increase at 18 years. Fig. 2 shows that the adjusted associations between score and PEs and PL score and depression increased in strength over time.

##### 3.2.1.2 ESL

Fig. 1c and d shows box plots of ESL scores against PEs and depression at both ages. There was little or no difference between median ESL scores between those who had suspected or definite PEs or depression and those who did not, at either age.

After adjustment there was no evidence of an association between ESL score and depression or between ESL score and PEs at either age. Fig. 2 shows that although the adjusted association between ESL and depression was slightly stronger than that with PEs at both ages, the confidence intervals included a null association in both cases. The strengths of the associations changed little over time.

#### 3.2.2 Psychopathological specificity

Table 2, Table 3 show the unadjusted and adjusted results of the likelihood ratio tests of the differences in the strength of the associations within each time-point across psychopathologies.

The unadjusted analyses in Table 2 show that the only risk factor with a specific association with both psychopathologies was gender and this was only true with associations at 12 years. The association between gender and depression at 12 was much stronger (a 61% increase in odds for women) compared to the association with PEs at 12 (a 14% increase in odds). There was marginal evidence of a psychopathologically specific effect with maternal education where having a mother with higher educational qualifications was associated with a 9% decrease in the odds of PEs at 12 years but only a 2% decrease in the odds of depression at the same age. In the adjusted associations in Table 3 only the association between PL score at 12 years showed evidence of psychopathological specificity. A 1 SD increase in PL score was associated with a 22% increase in the odds of PEs but only a 1% increase in the odds of depression.

### 3.3 Secondary analysis

#### 3.3.1 Differences in the associations between speech and language and depression and PEs over time

There was little evidence that the size of the associations between either PL or ESL and depression and PEs differed over time. In general the associations were slightly weaker at 18 years compared to 12 years for both psychopathologies. The differences over time were greater for the associations between each psychopathology and ESL, although the overlapping 95% confidence intervals at each time-point suggested no evidence of a true difference (results available on request).

## 4 Discussion

Our hypothesis that childhood PL problems, but not ESL problems would be associated with PEs was supported. Increasing levels of PL problems were associated with PEs at both 12 and 18 years. There was no evidence of an association between PEs and ESL at either age.

Our hypothesis that ESL problems but not PL problems would be associated with risk of later depression was not supported. There was no evidence of an association between ESL problems and depression at either age.

In general the effect sizes of the association between PL score and PEs were larger than between PL scores and depression, although this was more noticeable at 12 years. The associations between ESL scores and depression were very slightly stronger than those between ESL scores and PEs, and the difference was slightly larger at 18 years. However the overlapping confidence intervals surrounding the estimates at each time point show no evidence of a true difference.

There was also little evidence of any true change in the strength of the associations between speech and language ability and either psychopathology over time.

### 4.1 Previous research

Our findings support those of the only other study (Cannon et al., 2002) to investigate ESL and later PEs and the associations are similar in size (our findings - β = 0.06, 95% CI − 0.01, 0.31; Cannon et al. β = − 0.11, 95% CI − 0.42, 0.19). There have been no previous studies to investigate the association between PL and PEs.

Our study does not support the findings of the only other prospective study, of which the authors are aware, that examined ESL in childhood and internalising behaviour (believed to indicate depression (Archenbach and Rescorla, 2001)) in adolescence (Bornstein et al., 2013) which found an association. This study used a path analysis to investigate the association between speech and language development during childhood and early adolescence and both internalising and externalising behavioural problems in 224 participants.

### 4.2 Mechanisms

The measure of PL in this study reflects skills which are strongly associated with social cognitive ability. There is evidence that social cognitive deficits are associated with psychosis (Sprong et al., 2007) but conflicting evidence of the association with PEs, with some finding an association (Polanczyk et al., 2010, Thompson et al., 2011) but others not. There is also evidence of this association in those at high risk of psychosis (Thompson et al., 2013). The authors have previously found no evidence of an association in the same cohort between many of the domains of social cognition and PEs (Sullivan et al., under review), suggesting that the association found here between PL and PEs is not simply due to poor social cognitive ability.

Our findings also suggest that poor PL is also associated with late adolescent depression. The mechanism for this is not clear although there is recent evidence that poor social cognitive ability is associated with depression (Loi et al., 2013, Schreiter and Pijnenborg, 2013, Wolkenstein et al., 2011) and our findings suggest that a social cognitive deficit may precede depressive symptoms.

Poor speech and language ability in either or both domains may lead to more social isolation due to a reduced ability to communicate effectively with peers. Social isolation has previously been associated with depression (Joiner and Timmons, 2008, Kessler et al., 2003) and there is evidence (Sullivan et al., 2015) that poor relationships with peers precedes PEs. Social isolation can result in less “reality checking” with other people and therefore putative delusional thought processes can become entrenched.

### 4.3 Strengths and limitations

The large sample is an important strength and has enabled the detection of associations with reasonable precision and suggests that sampling bias is a less likely explanation for our findings.

The semi-structured interview assessment of PEs by trained interviewers is likely to have reduced the effect of measurement error and information bias in outcome measurement.

Multivariate probit modelling has allowed the direct comparison of the strength of associations between risk factors and outcomes and to investigate whether there are any true differences in associations both over time and between psychopathologies.

The imputation of missing data using a broad range of variables related to missingness or to the exposure or outcome has helped to minimise the possibility of selection bias resulting from the loss of participants from the cohort over time. In common with other cohorts ALSPAC participants were more likely to be lost to follow-up if they had PEs or high levels of depressive symptoms. Therefore, without imputation of missing outcome data to correct this the results would have been biased in the direction of a null finding.

It is not possible to be certain that deficits in speech and language ability definitely preceded PEs since there is no measure of PEs before age 9. However the interpretation of these experiences in very young children is unlikely to be reliable. The authors were only able to find one study where a very early assessment of PEs had been conducted (Scott et al., 2009b).

It is also possible that poor communication ability had an effect on the way that interviewers rated PEs. It is not possible to know whether this may have resulted in over or under-rating of the outcome.

While the measure of PL covered a wide range of language problems, that of ESL was more limited and focussed mostly on speech production and fluency with only four questions assessing syntax production. This may mean that the measure of ESL was less accurate which may have made a true association with PEs more difficult to detect.

Both speech and language measures were assessed by parental report which may not be an accurate reflection of the child's true ability. It is also possible that a parent with impaired PL or ESL may report on their child's speech and language ability in a different way from parents with normal PL and ESL abilities. Unfortunately we did not have access to any data on parental abilities in order to check this possibility. There may be an ethnic or cultural context to parental reporting of child speech and language abilities. The ALSPAC cohort has very few participants who are not white and British and therefore we expect this factor to have had little effect on our results.

## 5 Conclusion

It is important to identify childhood interventions which may prevent later psychopathology. Our findings suggest that poor PL in middle childhood can precede early and late adolescent PEs and late adolescent depression. If PL is a causal mechanisms of later disorder an effective intervention which improves this skill may reduce the risk. There is some evidence (Adams et al., 2012, Owens et al., 2008, Pierson and Glaeser, 2005) that it is possible to improve PL ability in children, suggesting that this may be a fruitful avenue to pursue. An example of an intervention that has been trialled (Messer et al., 1995) is LEGO therapy which encourages small groups of children to use social communication skills in a model building task. Other evidence based interventions include Comic Strip Conversations, Social Communication Intervention Programme and Social Stories (Trust, 2015).

## Role of funding source

The UK Medical Research Council (Grant ref: 74882), the Wellcome Trust (Grant ref: 076467) and the University of Bristol provide core support for ALSPAC. This study was funded by MRC grant G0701503.

## Contributors

Sarah Sullivan initiated and developed the research idea, conducted the analysis and wrote the paper. Linda Hollen and Yvonne Wren provided advice on existing literature as well as speech and language development in children and adolescents. Glyn Lewis and Stan Zammit provided expertise on psychiatric epidemiology and on psychopathology in children and adolescents. Andrew Thompson provided expertise on psychopathology in adolescents. All authors read and commented on drafts and approved the final version of the paper.

## Conflict of interest

None of the authors have any conflicts of interest to declare.

## Figures and Tables

**Table 1:** Descriptive data for study participants (n = 7659) including proportion of missing data and comparison between observed and imputed data.

Variable	Range/categories	Time point (years)	% of missing data	Observed values	Values post imputation
Psychotic experiences	Suspected/definite	18	38.4%	9.2%	9.4%
12	11.3%	13.7%	14.1%
Depression	Yes	18	41.3%	18.2%	18.8%
12	12.7%	5.3%	5.3%
Expressive speech and language score mean (SD)	96–162	9	20.8%	106.77 (7.43)	107.19 (7.54)
Pragmatic language score mean (SD)	46–70	25.3%	48.82 (2.11)	48.87 (2.13)
Gender	M		0%	47.8%	47.8%
Mother's marital status	Single	At birth	8.0%	14.3%	14.3%
Mother's educational status	A level/degree	9.4%	43.5%	42.6%
Non-verbal IQ mean (SD)	70–151	8	20.0%	100.50 (17.01)	98.29 (17.27)
Autistic traits mean total factor score	− 3.62 to 1.40	6 months–9 years	2.7%	0.03 (0.34)	0.04 (0.34)

**Table 2:** Unadjusted risk factor effects (OR and 95% CI) on depression and psychotic experiences (PEs) at 12 and 18 years and examination of whether disorder specific effects differ from a common effect at 12 and 18 years (n = 7659).

	Age (years)	Depression		PEs		Wald test
Common effect^b^ 12 years 18 years
OR	95% CI p	OR	95% CI p	p
*Confounding variables*						
Gender	12	1.61	1.37, 1.89 p ≤ 0.01	1.14	1.01, 1.28 p = 0.03	p ≤ 0.01
18	1.57	1.37, 1.79 p ≤ 0.01	1.33	1.13, 1.57 p ≤ 0.01	p = 0.10
Low maternal education	12	0.98	0.92, 1.05 p = 0.65	0.91	0.87, 0.96 p ≤ 0.01	p = 0.05
18	0.86	0.80, 0.91 p ≤ 0.01	0.85	0.80, 0.91 p ≤ 0.01	P = 0.74
Marital status of mother	12	0.95	0.90, 1.00 p = 0.08	0.86	0.82, 0.91 p ≤ 0.01	p = 0.17
18	0.91	0.87, 0.95 p ≤ 0.01	0.91	0.88, 0.95 p ≤ 0.01	p = 0.09
IQ at age 8	12	1.00	0.99, 1.00 p = 0.02	0.99	0.99, 1.00 p ≤ 0.01	p = 0.28
18	0.99	0.99, 0.99 p ≤ 0.01	1.00	0.99, 1.00 p = 0.04	p = 0.04

*Main risk factors*						
Pragmatic language at age 9^a^	12	1.59	1.44, 1.76 p ≤ 0.01	1.34	1.24, 1.44 p ≤ 0.01	p = 0.13
18	1.31	1.21, 1.41 p ≤ 0.01	1.28	1.16, 1.41 p ≤ 0.01	p = 0.87
Expressive speech and language at age 9^a^	12	1.27	1.16, 1.40 p = 0.02	1.11	1.04, 1.20 p = 0.01	p = 0.65
18	1.15	1.06, 1.24 p = 0.01	1.06	0.96, 1.17 p = 0.38	p = 0.18

a Per SD change.

b Tests evidence against the null hypothesis that there is no difference in the strength of the effect when each outcome is modelled separately compared to a model when depression and PEs are constrained to be equal at each time point; e.g. p ≤ 0.01 indicates very strong evidence that there is a true difference between the strength of the association between a risk factor and depression compared with the same risk factors and psychotic experiences.

**Table 3:** Main risk factor effects (OR and 95% CIs) on depression and psychotic experiences (PEs) at 12 and 18 years respectively, adjusted for confounding variables (n = 7659).

Main risk factors	Age	Depression		PEs		Wald test
Common effect^b^ at each time-point p
OR	95% CI p	OR	95% CI p
Pragmatic language^a^	12	1.01	0.89, 1.14 p = 0.87	1.22	1.11, 1.34 p ≤ 0.01	p = 0.01
18	1.10	1.00, 1.22 p = 0.05	1.25	1.10, 1.41 p ≤ 0.01	p = 0.11
Expressive speech and language^a^	12	1.02	0.91, 1.14 p = 0.78	1.02	0.91, 1.14 p = 0.68	p = 0.99
18	1.02	0.94, 1.11 p = 0.60	0.99	0.89, 1.10 p = 0.82	p = 0.61

a Per SD change.

b Tests evidence against the null hypothesis that there is no difference in the strength of the effect when each outcome is modelled separately compared with a model when depression and PEs are constrained to be equal at each time point; e.g. p ≤ 0.01 indicates very strong evidence that there is a true difference between the strength of the association between a risk factor and depression compared with the same risk factors and psychotic experiences.
